# Decoupling Oxygen Tension From Retinal Vascularization as a New Perspective for Management of Retinopathy of Prematurity. New Opportunities From β-adrenoceptors

**DOI:** 10.3389/fphar.2022.835771

**Published:** 2022-01-21

**Authors:** Luca Filippi, Maurizio Cammalleri, Rosario Amato, Massimiliano Ciantelli, Alessandro Pini, Paola Bagnoli, Massimo Dal Monte

**Affiliations:** ^1^ Department of Clinical and Experimental Medicine, University of Pisa, Pisa, Italy; ^2^ Department of Biology, University of Pisa, Pisa, Italy; ^3^ Department of Experimental and Clinical Medicine, University of Florence, Florence, Italy

**Keywords:** hyperoxia/hypoxia, hypoxia-inducible factor-1, β-adrenergic system, propranolol, prolyl hydroxylase domain-containing proteins

## Abstract

Retinopathy of prematurity (ROP) is an evolutive and potentially blinding eye disease that affects preterm newborns. Unfortunately, until now no conservative therapy of active ROP with proven efficacy is available. Although ROP is a multifactorial disease, premature exposition to oxygen concentrations higher than those intrauterine, represents the initial pathogenetic trigger. The increase of oxygenation in a retina still incompletely vascularized promotes the downregulation of proangiogenic factors and finally the interruption of vascularization (ischemic phase). However, the increasing metabolic requirement of the ischemic retina induces, over the following weeks, a progressive hypoxia that specularly increases the levels of proangiogenic factors finally leading to proliferative retinopathy (proliferative phase). Considering non-modifiable the coupling between oxygen levels and vascularization, so far, neonatologists and ophthalmologists have “played defense”, meticulously searching the minimum necessary concentration of oxygen for individual newborns, refining their diagnostic ability, adopting a careful monitoring policy, ready to decisively intervene only in a very advanced stage of disease progression. However, recent advances have demonstrated the possibility to pharmacologically modulate the relationship between oxygen and vascularization, opening thus the perspective for new therapeutic or preventive opportunities. The perspective of a shift from a defensive towards an attack strategy is now at hand.

## Introduction

Retinopathy of prematurity (ROP) is a potentially blinding ocular disorder characterized by anomalous blood vessel growth in the retina of premature infants (Hellström et al., 2013). During the intrauterine fetal life, retinal vessels development begins between the 10th and the 15th week of gestation and stops at term, when vessels reach the retinal periphery, approximately at the 40th week (Hughes et al., 2000). Therefore, in premature infants born with very low gestational age, the retina is only minimally vascularized, and this incomplete vascularization represents the anatomical prerequisite for the development of ROP.

Although ROP is clinically diagnosed in infants when an abnormal vascular proliferation of retina begins, the pathophysiologic conditions favoring the disorder develop much earlier, probably starting since the first days of life. In the first 4–5 weeks of extrauterine life, the vascularization process is interrupted or even regresses (ischemic phase of ROP) thus leaving the peripheral retina avascular until an approximate postmenstrual age of 32 weeks. However, the persistent ischemia together with the increased metabolic requirements of the developing retina transforms the disease within a few weeks: in fact, a progressive hypoxia develops in the outermost areas of the retina triggering its neo-vascularization, sometimes tumultuously (proliferative phase of ROP). The degree of ischemia in the first weeks of life influences then the extent of hypoxia and finally the tumultuousness of the subsequent neovascularization. The aggressiveness of the disease depends on the retinal area progressively covered by proliferating vessels, on the vascularization stage (from stage 1 to 5), and on the presence of dilatation and tortuosity of the posterior pole vessels (ROP plus disease) (Table 1) (Chiang et al., 2021). In the more advanced stages of the disease, vascular proliferation can induce the development of intravitreal fibrosis with the consequent retinal traction and detachment (Hellström et al., 2013). This is the reason why, despite of progressive improvements in neonatal care, ROP still represents the principal cause of preventable childhood blindness and visual impairment throughout the world, in both developing and developed countries (Prakalapakorn et al., 2021).

**TABLE 1:** ROP classification (Chiang et al., 2021).

Location of vascularization		
Zone I	Twice radius from optic disc to the fovea	
Zone II	From the outer border of Zone I to the nasal ora serrata	
Zone III	Residual peripheral retina extending beyond Zone II	
Stage of the disease		
Stage of acute disease	Stage 1	Demarcation line at the vascular-avascular juncture
	Stage 2	Ridge from the demarcation line with small neovascular tufts
	Stage 3	Neovascular proliferation from the ridge into the vitreous or flat neovascularization
Retinal Detachment	Stage 4	Partial detachment: 4A with fovea attached, 4B with fovea detached
	Stage 5	Total detachment
Preplus disease	Abnormal vascular dilation, tortuosity insufficient for plus disease, or both	
Plus disease	Appearance of dilation and tortuosity of retinal vessels	

ROP affects preterm newborns usually born before 31 weeks of gestation and with very low birth weight (VLBW) weighing less than 1,500 g, even though the vast majority of severe ROP cases have been found in infants with lower gestational age and weight at birth (Quinn et al., 2018). Approximately 32–37% of VLBW hospitalized infants in the Neonatal Intensive Care Units participating to the largest and most comprehensive databases of high-risk infants such as the Vermont Oxford Network, is affected by ROP, consistent with most studies over time. However, ROP shows a variable clinical expression that can progressively evolve towards stages of greater aggressiveness and risk for visual function. The rate of progression to the most severe stages of disease has been reported to be approximately a quarter of newborns with ROP (Chang, 2019; Filippi et al., 2019; Prakalapakorn et al., 2021). In future, prevalence of severe vision loss and blindness due to ROP is expected to increase drastically with a major involvement of Asian countries that account for half of the world’s preterm birth and ROP-related blindness (Sen et al., 2020).

ROP is a multifactorial disease, where prenatal, perinatal, and neonatal factors play an important role in the perturbation of the normal vascular development of the retina. For instance, despite no specific genetic variants have been associated with ROP occurrence (Swan et al., 2018), evidence exists for a possible hereditary predisposition. In this respect, many studies have demonstrated that African Americans infants have a lower incidence of threshold ROP (defined as disease with a 50% likelihood of progressing to retinal detachment) (Saunders et al., 1997; Yang et al., 2006; Port et al., 2014). Besides genetic predisposition, intrauterine inflammation including chorioamnionitis has also been demonstrated to be an important risk factor for ROP development (Villamor-Martinez et al., 2018). The strong correlation with intrauterine infections has suggested the hypothesis that already during the prenatal life the development of infections can trigger a very precocious phase of ROP by interfering with retinal vascularization (Dammann et al., 2021). In addition, some postnatal comorbidities of preterm infants, including bacterial (Tolsma et al., 2011; Lee and Dammann, 2012) or fungal sepsis (Noyola et al., 2002; Huang et al., 2019) may accelerate or aggravate ROP progression. Similarly, necrotizing enterocolitis, one of the most serious gastrointestinal emergencies in preterm infants, increases the risk of developing severe forms of ROP requiring surgical treatment (Fundora et al., 2021).

Alongside the numerous clinical conditions that seem to favor the occurrence and the progression of ROP, prematurity and premature exposure to high oxygen tension by still immature retinal vascularization represent the two factors playing a determinant role (Kim et al., 2018).

## Role of Prematurity and Early Oxygen Exposure

It is well-known that the development of vascularization in the human retina needs a physiologically hypoxic environment, such as the intrauterine one (Hughes et al., 2000). The embryonic and fetal vascular development depends on the modulation of a series of proangiogenic factors, such as vascular endothelial growth factor (VEGF), mainly controlled by the hypoxia-inducible factor-1 (HIF-1) (Haigh, 2008; Krock et al., 2011). HIF-1 is a dimeric transcription factor composed by an α and a β subunits. While HIF-1β is stable, in normoxic conditions HIF-1α is hydroxylated by prolyl hydroxylase domain-containing proteins (PHDs), ubiquitinated by the von Hippen-Lindau protein and finally degraded by the proteasome. In hypoxia, PHDs are inactive, then HIF-1α escapes degradation, accumulates into the cell, migrates into the nucleus and, after dimerization with HIF-1β, activates the transcription of a plethora of oxygen-sensing genes (Wang and Semenza, 1993; Semenza, 2004). In light of the strict link between oxygen and vascularization, exposure to abnormal oxygen tensions along the developmental stages could drastically impact the physiological formation of vascular network in immature organs.

**FIGURE 1:**
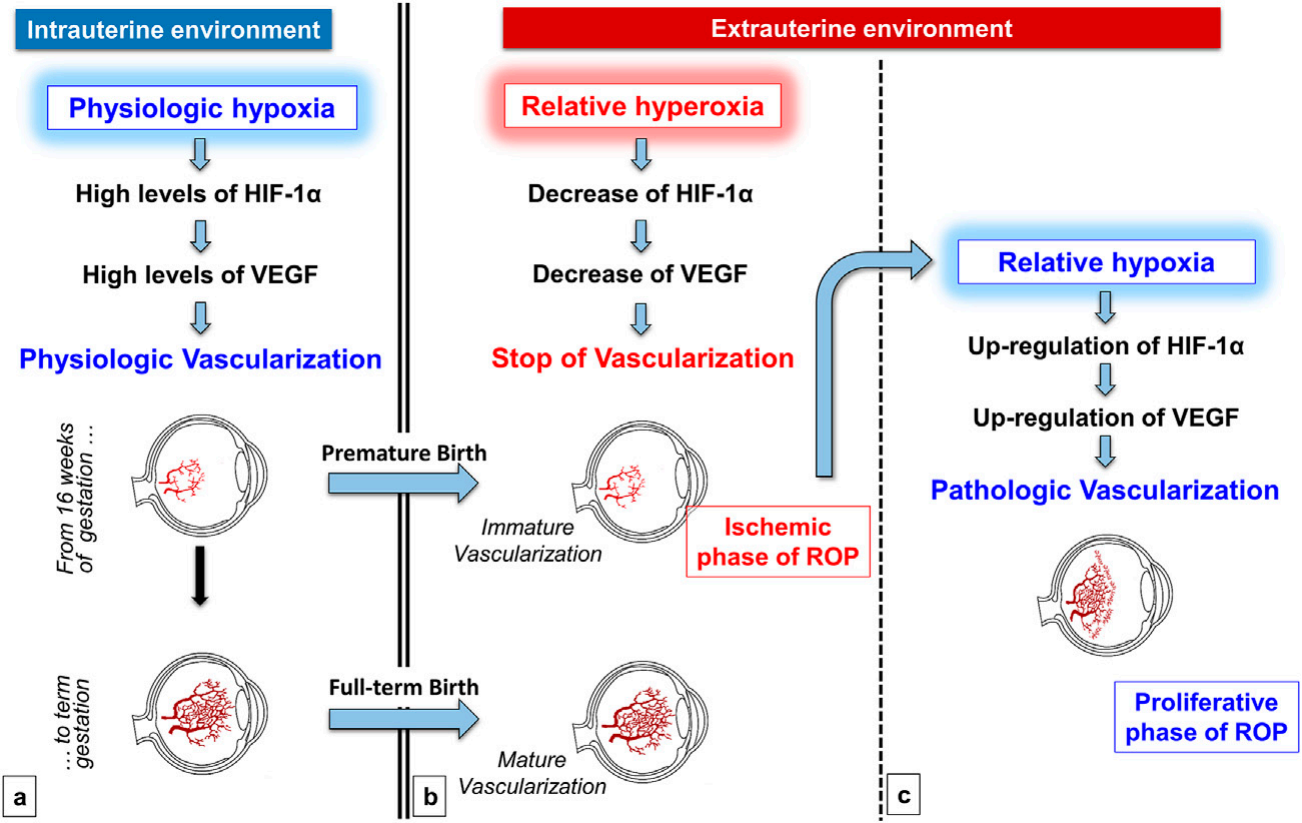
Role of oxygen in the physiologic retinal vascularization and in the pathogenesis of ROP. During the intrauterine life, the physiologic low tension of oxygen promotes Hypoxia-Inducible Factor-1α (HIF-1α) and consequently Vascular-Endothelial Growth Factor (VEGF) upregulation, favoring the physiologic vascularization of retina **(A)** Premature oxygen exposition of preterm newborns induces the stop or even the regression of the immature retinal vascularization, secondary to the downregulation of both HIF-1α and VEGF (ischemic phase of ROP) **(B)** This progressive ischemia is responsible for the shift towards a retina that progressively becomes again hypoxic. Retinal hypoxia in turn induces HIF-1α and VEGF upregulation that promote a tumultuous and pathologic retinal neovascularization (proliferative phase of ROP) **(C)**.

In this respect, according to the classic interpretation of ROP pathogenesis, both the ischemic and the proliferative phases of ROP are driven by abnormal oxygenation within the immature retina, hyperoxia for the first phase and hypoxia for the second phase (Hellström et al., 2013). In particular, the birth of a premature newborn inevitably implicates an early exposure to an oxygen tension of at least 21% or even higher in case of respiratory insufficiency. The premature exposition to a relatively hyperoxic environment promotes the interruption or regression of retinal vascularization. Ophthalmologists define this phase as “*incomplete vascularization*” (Chiang et al., 2021), and it is clinically impossible to predict at this stage whether the evolution will be towards the normal vascularization or the development of ROP. The evolution towards an anomalous blood vessel growth typical of ROP depends on the degree of ischemia leading consequently to hypoxia in the avascular districts (Figure 1).

These events are well mimicked by the mouse model of oxygen-induced retinopathy (OIR) (Smith et al., 1994), a model used extensively to mirror ROP, which shows the biphasic course of the disease. On the seventh day of life, newborn pups, whose retinal vascularization is normally immature, are exposed for 5 days to hyperoxia, which down-regulates the production of proangiogenic factors and promotes a wide vaso-obliteration around the optic nerve head (ischemic phase). This phase mimics what happens to preterm newborns prematurely exposed to a relatively hyperoxic environment. At the end of the 5 days, newborn pups are returned to a normoxic environment for further 5 days. This sudden reduction in oxygenation is perceived by the mouse retina as a relative hypoxia, thus inducing HIF-1-mediated neovascularization. (Smith et al., 1994). This vascular sprout in the mouse mimics the tumultuous vascularization observed in human infants when the increasing metabolic requirement of the ischemic retina induces a progressive marked hypoxia. Intense vascular proliferation represents a serious risk of hemorrhages and vitreous edema because neovessels are still immature and hyperpermeable (Vähätupa et al., 2020). In humans the consequences of this progression can be significantly more dramatic because fibrosis and consequent retinal tractions can result in retinal detachments.

The pathogenic role of oxygen is indirectly confirmed by clinical studies addressed to establish the ideal range of oxygen saturation (SpO2) for preterm infants. Two large randomized-controlled studies comparing two different ranges of SpO2 (85–89% vs. 91–95%) showed that the higher SpO2 range, although improving infants’ survival, induced a significant higher incidence of severe ROP (SUPPORT Study Group et al., 2010; BOOST-II Australia et al., 2016), despite this eventuality remains controversial (Schmidt et al., 2013). Therefore, the increased ROP incidence appeared an unavoidable consequence of a reduced mortality obtained with higher oxygen levels. This conclusion has been recently confirmed by a meta-analysis which established that the assignment to the higher SpO2 range ensures a reduced risk of additional life-threatening complications, even though at the expense of a significant increased risk of severe ROP (Askie et al., 2018). This is the reason why the Committee on Fetus and Newborn of the American Academy of Pediatrics suggests a levels of 90–95% as the ideal target range of SpO2 (Cummings et al., 2016).

In conclusion, preterm newborns seem to have a paradoxical relationship with oxygen, which on the one hand guarantees and improves their survival, but on the other hand represents the signal that interrupts the vasculogenic processes and therefore lays the foundations for the development of ROP.

## Current Strategies for the Management of ROP

The strategy currently adopted for ROP management is limited to the scrupulous monitoring of ROP development and progression with the aim of identifying the high-risk vascular abnormalities in the developing retina but without the actual possibility to significantly interfere with the disease occurrence and progression. In effect, substantial intervention protocols are currently available almost exclusively for the advanced stages of ROP aiming at minimizing the effects of vascular proliferation (Fierson, 2019).

### Current Strategies for Advanced ROP Treatment

Cryotherapy (the cold burn of the total thickness of the immature avascular retina) was the first treatment that was adopted for severe ROP (Multicenter trial, 1988). This treatment was afterwards replaced by the less painful and less harmful laser photocoagulation (Connolly et al., 2002; Ng et al., 2002), which currently represents the standard therapy for the most advanced stages of ROP (Hellström and Hard, 2019). However, this approach is not free from adverse effects and complications. Adverse effects include the need of anesthesia for infants and specific skills of the ophthalmologist. Moreover, around 10% of cases require repeated intervention, depending on the ophthalmologist experience (Early Treatment For Retinopathy Of Prematurity Cooperative Group et al., 2003). Finally, laser treatment can produce some visual dysfunction, such as loss of the visual field, high myopia, intraocular hemorrhage, corneal oedema, intraocular pressure increase (Hurley et al., 2006). Further eye complications include corneal burns, band keratopathy, hyphaemia, and cataract (Kieselbach et al., 2006). The intravitreal injection of neutralizing anti-VEGF drugs currently represents an alternative treatment that avoids retinal destruction, even though it is not free from adverse events and complications. In addition, short- or long-term safety profile of anti-VEGF drugs is still under investigation, and further studies are required to evaluate their effects on retinal developmental processes (Sankar et al., 2018). The first prospective, randomized, controlled trial comparing the intravitreal anti-VEGF bevacizumab to conventional laser therapy reported an increased efficacy of anti-VEGF as compared with laser therapy for stage 3 plus ROP, but only in Zone I (Mintz-Hittner et al., 2011). Since this first study, the efficacy of anti-VEGF drugs in comparison to laser photocoagulation has been the subject of extensive debate. In respect to laser photocoagulation, anti-VEGF drugs have been recently reported to display higher retreatment incidence although less burdened by eye complications and increased myopia (Li et al., 2018). On the other hand, a decreased incidence of retinal detachment has been evidenced in anti-VEGF treated patients, probably due to the fact that VEGF levels decrease faster after anti-VEGF drugs than after laser therapy (Barry et al., 2021). In addition, a reduced rate of optic atrophy and amblyopia has been observed in patients treated with anti-VEGF drugs presumably due to improved foveal development as compared to laser photocoagulation (Gundlach et al., 2021).

The current prevalent therapeutic strategy is predominantly focused on the careful screening and early diagnosis of ROP, in the strict monitoring of its evolution, but the therapeutic interventions are planned only in an advanced phase of the disease.

### Current Strategies to Counteract ROP Progression

The strict relationship between high oxygen level and reduced retinal vascularization, suggested to counteract ROP progression by administering additional oxygen during the proliferative phase of the disease. However, results of studies that adopted this strategy are discordant. In the first explorative studies, newborns with ROP and managed with two SpO2 targets (approximately 89–94 versus 95–99%) showed a similar percentage of ROP progression to the threshold for ablative retinal surgery (Supplemental, 2000; Askie et al., 2003), even though some benefits were reported for infants in the higher SpO2 arm who showed a lower incidence of plus disease (Supplemental, 2000). An additional retrospective cohort study demonstrated that the increase of SpO2 target over 97% reduced ROP progression (Colaizy et al., 2017). Finally, a more recent retrospective study suggested a “biphasic strategy” with a lower SpO2 target for the first weeks of life, when oxygen usually induces retinal ischemia, and a target over 95% after the 34th week, when a reduction of angiogenic processes is desirable, as a probably best approach to prevent ROP (Shukla et al., 2019).

Therefore, one of the most promising approaches to counteract ROP progression appears again related with a careful modulation of oxygen administration, because oxygen supplementation is indissolubly associated with the suppression of HIF-1α-induced angiogenic cascade. However, the higher oxygenation target eventually limiting the severity of ROP would also impact with additional immature districts such as brain regions (Reich et al., 2016) or lungs (Balasubramaniam et al., 2007), which are very sensitive to oxygen levels.

At the end, modulating oxygen levels does not appear a winning strategy to alleviate ROP severity, but, unfortunately, until now no conservative treatments with proven efficacy to slow, stop or reverse ROP progression are currently available. Therefore, there is an urgent need for new strategies aimed to counteract ROP progression or even to prevent its occurrence.

### Current Strategies to Prevent ROP Occurrence

In consideration of the strong relationship with prematurity, the best policies to reduce ROP occurrence are based on the prevention of preterm delivery, the improvement of antenatal and perinatal care, together with the adoption of neonatal protocols for nursing premature infants. In this regard, the administration of prenatal steroids to high-risk mothers delivering preterm newborns is a proven approach to prevent a plethora of neonatal complications, including ROP (Yim et al., 2018). In addition, the implementation of protocols to reduce neonatal infections, to encourage the use of human milk (Zhou et al., 2015) together with other supportive care which can be ensured by an adequate nursing setting (Darlow et al., 2013) represent some of the strategies currently adopted to reduce ROP occurrence. Among many strategies, the appropriate oxygen management currently constitutes the most effective mean to prevent ROP occurrence or to mitigate ROP severity.

So far, neonatologists, aware of the bivalent effect of oxygen, able to guarantee a better survival expectancy but also to increase the risk of ROP, have “played defense”, meticulously searching the “minimum necessary concentration” of oxygen for individual newborns, mainly during the first weeks of life (Manja et al., 2017). Much progress has been made, above all avoiding the unnecessary use of oxygen. In this respect, among the many initiatives, neonatal resuscitation is now initiated with the minimum amount of oxygen strictly necessary (Kim and Nguyen, 2019), newborns are frequently ventilated using automated control system, developed to rapidly modulate the fraction of inspired oxygen (Mitra et al., 2018), even though the results are still uncertain (Salverda et al., 2021). This attitude is based on the lack of current strategies aimed to uncouple the relationship between oxygen levels and vascularity.

## New Perspectives to Counteract ROP Progression

In light of the pitfalls and limitations of the current strategies for the management of ROP, it appears increasingly evident that the strategies not intended to decouple oxygen levels from vascular effects, as those adopted so far, seem destined to fail. Therefore, both the possibility to early intervene on the ROP occurrence and progression and the improvement of the advanced ROP management are likely to be attainable adopting a decoupling strategy.

As previously mentioned, within the complex interplay of molecular mechanisms coupling oxygen levels to vascular adaptive mechanisms, HIF-1 represents the primary biological sensor of tissue oxygenation. Therefore, the pharmacological modulation of HIF-1 and its subsequent desensitization from oxygen appears as the most immediate and reasonable approach to decouple oxygen from ongoing vascular processes. In this respect, several preclinical evidence from the OIR model have highlighted the actual possibility to pharmacologically interfere with the oxygen-dependent HIF-1 transcriptional activity by inhibiting its PHD-dependent degradation during the hyperoxic stage or inhibiting its activity during the hypoxic stage. In fact, the systemic administration of competitive PHD inhibitors, such as dimethyloxalylglycine or Roxadustat, during the hyperoxic stage of OIR has been shown to reduce the vascular regression by increasing the levels of HIF-1α and pro-angiogenic factors such as VEGF and erythropoietin, thus resulting in the amelioration of the vascular tufting and tortuosity in the subsequent hypoxic stage (Sears et al., 2008; Hoppe et al., 2016). Similarly, the administration of HIF inhibitors such as topotecan, suppressing the translation of HIF-1α, or doxorubicin, blocking HIF-1 binding to the hypoxia-response element, during the hypoxic stage of OIR has been shown to decrease the pathological vascular proliferation by inhibiting the surge of pro-angiogenic factors (Miwa et al., 2019).

Moreover, a series of PHD inhibitors and HIF-1 inhibitors are currently in clinical use for systemic diseases such as anemia (Joharapurkar et al., 2018) or for cancer therapy (Shirai et al., 2021). Despite the efficacy of this approach, demonstrated by pre-clinical studies, and the current availability of drugs in clinics, concerns exist regarding the HIF-1 modulation, especially in immature infants displaying ongoing developmental processes. In effect, the modulation of a transcription factor regulating a wide range of genes could significantly increase the risk of severe side effects including cancer, thromboembolism, pulmonary hypertension or hyperkaliemia (Hirota, 2021). For this reason, the modulation of alternative pathways involved in the oxygen-vascularization coupling are needed for a safer and more realistic translation to the clinical practice.

### Treatment With Propranolol

In the complex relationship between hypoxia and neovascularization, studies performed in preclinical models of ROP over the last decade have suggested that, together with HIF-1, a significant role is covered by the β-adrenergic system (Casini et al., 2014). Preliminary indications of its involvement in the oxygen-dependent vascular processes have derived from evidence demonstrating that the levels of both noradrenaline (NA) and its receptors are increased by hypoxic conditions. In this respect, NA increased significantly in isolated perfused pulmonary canine arteries when nerves are stimulated under hypoxia (Rorie and Tyce, 1983) and in plasma of human after long residence at more than 5,000 m of altitude (Calbet, 2003). At the meantime, β2-adrenoceptor (β2-AR) overexpression has been demonstrated in the ischemic rat femoral artery in which upregulated β2-ARs trigger VEGF-induced endothelial cell proliferation (Iaccarino et al., 2005) or in a rat model of post-myocardial infarction heart failure, driving VEGF-induced endothelial proliferation (Galasso et al., 2013).

The proangiogenic role played by the β-adrenergic system may explain why severe ROP occurs less frequently among African American infants, who have a higher incidence of a polymorphism of G protein-coupled receptor kinase 5 (Liggett et al., 2008). This genetic variant favors β-AR desensitization, thus leading to resistance to noradrenergic stimulus, explaining why African American infants are protected against ROP severity (Good et al., 2012; Good, 2020).

However, most of the information regarding the role of β-ARs in hypoxia-induced neovascularization originates from the fortuitous demonstration that the progression of infantile hemangiomas (IHs) is effectively reduced by a treatment with propranolol (an unselective β1-and β2-AR antagonist) (Léauté-Labrèze et al., 2008). Until then, little was known about the pathogenesis of IHs, except for the role played by local hypoxia, considered as the stimulus capable of triggering reactive vascular proliferation (Drolet and Frieden, 2010). More recently, a variety of studies demonstrated that the efficacy of propranolol is mainly related to the blockade of β2-ARs and the consequent inhibition of proangiogenic factors such as VEGF (Ji et al., 2013). In light of these findings, propranolol administration has become the treatment of choice for IHs, although the success rate of this treatment is about 60–70% (Léauté-Labrèze et al., 2015).

The antiangiogenic effects of propranolol aroused interest regarding the possible involvement of the β-adrenergic system in the pathogenesis of other human neonatal diseases similarly characterized by angiogenic processes induced by a hypoxic environment, such the proliferative phase of ROP. The similarities between the pathogenesis of IH and of ROP, including the role played by hypoxia-induced proangiogenic factors, suggested to explore the possible efficacy of a treatment with propranolol also for reducing ROP progression (Filippi et al., 2010).

The efficacy of this approach was at first demonstrated in the OIR mouse model. In this model, the administration of propranolol during the hypoxic phase of the disease reduced retinal neo-vascularization by preventing HIF-1α upregulation and its proangiogenic cascade (Ristori et al., 2011). The efficacy of propranolol was attributable to the β2-AR blockade since similar anti-angiogenic effects were obtained by administering the β2-AR selective antagonist ICI 118,551, but not after the administration of the β1-AR selective antagonist atenolol (Martini et al., 2011). The hypothesis that retinal neovascularization would result from a combined interaction between ligands and receptors was supported by the demonstration that in the OIR model, during the proliferative phase, NA is significantly upregulated (Dal Monte et al., 2012) and drives an overstimulation of β2-ARs, which are mainly expressed by Müller cells. Overstimulated β-adrenergic system induces VEGF upregulation and consequently neovascularization, that is efficiently counteracted by β2-ARs antagonism (Martini et al., 2011).

Besides to the efficacy on retinal angiogenesis, propranolol could be expected to exert a neuroprotective effect. Even though ROP is usually considered a vascular disease, noninvasive electroretinogram (ERG) performed in ROP patients show functional visual deficits attributable to a dysfunction of photoreceptors and post-receptor retinal neurons (Hansen et al., 2017). Similar impairment can also be detected in OIR models in which an altered ERG has been recorded as a consequence of neuronal cell suffering and death (Fulton et al., 2009). In this respect propranolol, in the mouse OIR model, was found to counteract visual dysfunction by protecting retinal cells through the direct modulation of survival mechanisms such as the stimulation of autophagy and the inhibition of apoptosis (Cammalleri et al., 2017). A recent study demonstrated that, in a rat model of OIR, topical propranolol not only reduced retinal vascular damage but also prevented astrocytes degeneration (Qadri et al., 2021), suggesting that propranolol may play an indirect neuroprotective effect if one considers the relevant role played by astrocytes in retinal function. The neuroprotective properties of propranolol together with its anti-angiogenic activity during the hypoxic stages indicate an actual efficacy of the drug. In general, treatments reducing retinal vascular pathology may be expected to ameliorate neuronal defects that characterize OIR. However, there is also evidence indicating that treatments preventing pathological angiogenesis may not necessarily improve retinal function (Hatzopoulos et al., 2014). Experimental results of preclinical studies that evaluated the role of β-ARs in the OIR model are summarized in Table 2.

**TABLE 2:** Role of β-ARs in preclinical studies.

Preclinical studies			
References	OIR model	Treatment	Results
Ristori et al. (2011)	Mouse strain C57BL/6J	Subcutaneous PROP	Reduction of HIF-1α, VEGF, IGF-1, retinal hemorrhage, vascular tufts, capillary leakage
Martini et al. (2011)	Mouse strain C57BL/6J	Subcutaneous ICI 118,551	Reduction of VEGF, IGF-1, retinal hemorrhage, vascular tufts, capillary leakage, prevention dysfunctional ERG
Dal Monte et al. (2012)	Mouse strain C57BL/6J	Subcutaneous ISO	Reduction of VEGF, vascular tufts, desensitization of β2-ARs
Chen et al. (2012)	Mouse strain 129S6	Subcutaneous, oral or intraperitoneal PROP	No effects
Dal Monte et al. (2013a)	Mouse strain C57BL/6J	Topical PROP	Reduction of HIF-1α, VEGF, IGF-1, vascular tufts
Cammalleri et al. (2017)	Mouse strain C57BL/6J	Subcutaneous PROP	Reduction of apoptosis and stimulation of autophagy, prevention of dysfunctional ERG
Qadri et al. (2021)	Rat strain Sprague-Dawley	Topical or oral PROP	Improvement of vascular damage, prevention of astrocyte degeneration

PROP: propranolol; ISO: isoproterenol; HIF-1α: hypoxia-inducible factor-1α; VEGF: vascular endothelial growth factor; IGF-1: insuline-like growth factor; ERG: electroretinogram

Based on these preclinical studies, the efficacy and the safety of a treatment with propranolol, administered during the proliferative phase, was tested in human preterm newborns with ROP in a series of pilot clinical trials (Filippi et al., 2013; Makhoul et al., 2013; Bancalari et al., 2016; Korkmaz et al., 2017; Sanghvi et al., 2017; Sun et al., 2018; Ozturk and Korkmaz, 2019). Three recent meta-analyses demonstrated that oral administration of propranolol to newborns with ROP slows down the progression of retinal neovascularization and reduces the indication for laser photocoagulation or anti-VEGF drugs (Kaempfen et al., 2018; Stritzke al., 2019; Kong et al., 2021). However, treatment with propranolol aroused safety concerns because its systemic administration was responsible of life-threatening events in instable preterm infants (Filippi et al., 2013). In order to prevent adverse effects, topical administration of propranolol was explored, with the objective to obtain similar efficacy of oral approach, but with lower plasma concentrations. Also in this case, animal experiments preceded human exploration. Firstly, in OIR mice, propranolol eye-drops were demonstrated to be effective in counteracting retinal neovascularization (Dal Monte et al., 2013a). Then, in healthy rabbits, the administration of 0.1% eye-drops promoted a dramatical increase of retina/plasma propranolol ratio if compared with oral administration, demonstrating the feasibility of topical approach (Padrini et al., 2014). Until now, two explorative clinical trials have demonstrated that the administration of eye micro-drops containing propranolol at either 0.1% or 0.2% in human infants was safe, even though only the higher concentration appeared to be effective in reducing ROP progression to the stages 2–3 with plus disease, from 23.7% of the historical control group to the 12.4% (Filippi et al., 2017, 2019). The low plasma concentration of propranolol and the lack of adverse effects in both studies encourage the administration of higher dose. This is the reason why a new trial with propranolol 0.4% eye micro-drops is planned (EudraCT number 2021-000131–31). The realistic objective of the treatment with propranolol is to obtain a reduction of ROP progression of about 60%, as is at the moment described by recent meta-analyses that have evaluated the few and small trials until now concluded (Kaempfen et al., 2018; Strizke al., 2019; Kong et al., 2021) and in line with the efficacy observed in the treatment of IHs (Léauté-Labrèze et al., 2015).

In spite of preclinical and clinical promising data, many questions still remain unanswered. First of all, it is crucial to irrefutably demonstrate that propranolol is able to significantly counteract ROP progression because clinical trials until now completed are not large enough to draw definitive conclusions. For this reason, further large, well-designed randomised trials are warranted to confirm or refute the role of propranolol (Kaempfen et al., 2018). Moreover, it is not yet definitively established which is the best route of propranolol administration (systemic or topic) as well as the most effective dose is not proven. The timing of the administration, conversely, seems more accurately identified as propranolol seems to exert protective effects only if administered during the proliferative phase and not prophylactically during the avascular phase. In fact, a recent trial demonstrated that preterm infants receiving propranolol treatment for cardiac dysfunction before ROP occurrence developed a dramatically severe retinopathy (Filippi et al., 2019). In this respect, precocious administration of propranolol, such as in the ischemic phase, would be detrimental when VEGF levels are downregulated thus preventing a normal retinal vascularization. In contrast, propranolol administration during the proliferative phase, when VEGF is upregulated and supports a tumultuous and dangerous neovascularization, would be suitable to reduce the undesirable effects of VEGF. In the meanwhile, considering the success rate observed in infants treated with propranolol for IHs or for ROP, attention should also be paid to patients unresponsive to propranolol. In this regard, it is right to wonder whether propranolol is the β-blocker more appropriate to counteract the proliferative phase of ROP. This statement implies the question of whether only β1-and β2-ARs are implicated in ROP neo-vascularization, or whether other receptors activated by NA surge are involved in hypoxia-induced vascularization (Filippi et al., 2015).

The possibility to effectively treat infants with ROP using propranolol is of great importance, as one of the most disabling prematurity-related complications may be counteracted with a cheap, readily available and widely known drug. In addition, the regression of IHs or ROP by propranolol suggests that the blockade of β-adrenergic system may participate to uncoupling hypoxia from vascularization, thus indirectly indicating that oxygen-induced modulation of vascularization processes involves at least in part the β-adrenergic system.

Clinical trials that explored the efficacy of propranolol treatment in infants with ROP are summarized in Table 3.

**TABLE 3:** Role of propranolol in clinical studies.

Clinical studies Randomized controlled trials						
References	Phase of ROP	Treatment until complete vascularization	Sample size intervention	Control	Sample size control	Results
Filippi et al. (2013)	Proliferative/Stage 2	Oral PROP 1–2 mg/kg/day	25	Standard treatment	26	Progression of ROP stage 61/185 vs 99/198 RR 0.65 (95% CI 0.47–0.88) Plus disease 11/134 vs. 27/147 RR 0.43 (95% CI 0.22–0.82) Kong et al. (2021)
Makhoul et al. (2013)	Proliferative/Stage 1–2	Oral PROP 0.5–2 mg/kg/day	10	Sucrose	10	
Sanghvi et al. (2017)	Ischemic	Oral PROP 1 mg/kg/day	51	Calcium carbonate	51	
Sun et al. (2018)	Proliferative/Stage 2	Oral PROP 0.5 mg/kg/day	41	Saline	43	
Ozturk and Korkmaz, (2019)	Proliferative/Stage 0–2	Oral PROP 2 mg/kg/day	58	Saline	68	
Other non-randomized controlled trials						
References	Phase of ROP	Treatment until complete vascularization	Sample size intervention	Control	Sample size control	Results
Bancalari et al. (2016)	Proliferative/Stage 2–3	Oral PROP 1.5 mg/kg/day oral	20	Historical control group	27	Progression to stage 2 or 3 with plus 2/20 vs. 13/27
Filippi et al. (2017)	Proliferative/Stage 2	Topical PROP 0.1%	23	Historical control group	26	Study discontinued
Filippi et al. (2019)	Proliferative/Stage 1	Topical PROP 0.2%	97	Historical control group	333	Progression to stage 2 or 3 with plus 12/97 vs. 79/333 RR 0.521 (95% CI 0.297–0.916)

PROP: propranolol; RR: Relative Risk (treatment vs. control); CI: confidence intervals

### A Futuristic Scenario

As outlined by different meta-analysis of randomized controlled trials, the efficacy of propranolol in reducing the progression of ROP is about 60% (Kaempfen et al., 2018; Strizke al., 2019; Kong et al., 2021) an effect comparable to that observed against IHs (Léauté-Labrèze et al., 2015). A such percentage is relevant and justifies why propranolol in a few years has become the gold standard in the treatment of IHs (Kowalska et al., 2021) and why there are so many expectations in the early treatment of infants with ROP. However, in ROP as in IHs, the presence of a similar percentage of non-responders suggests that receptors additional to β1-and β2-ARs may be involved in the pathogenesis of both diseases, possibly still belonging to the adrenoceptor family and activated by NA surge. Interestingly, the evaluation of β-AR expression in biopsy samples of IHs has recently shown that patients unresponsive to propranolol displayed significantly higher expression of β3-ARs (Bassi et al., 2021), the third members of the β-ARs family sharing 40–50% sequence homology with β1-ARs and β2-ARs but with main differences in the third intracellular loop and C-terminal tail (Granneman et al., 1993; Michel et al., 2020; Nagiri et al., 2021). This evidence could represent a possible indication that propranolol may be less effective when β3-AR expression is prevalent. In the retina, β3-AR is usually localized to the vascular endothelium and its expression has appeared strictly related with the tissue oxygenation. In effect, in the OIR model, β3-ARs were significantly upregulated in response to hypoxia and densely localized to the engorged vascular tufts of the inner capillary network (Ristori et al., 2011). Although the increment in β3-ARs during the hypoxic phase could be considered as an epiphenomenon due to the drastic proliferation of retinal vessels, some evidence have suggested the possible active involvement of β3-ARs in the vascular proliferation: 1) the strict relationship between β3-ARs upregulation and hypoxia had already been demonstrated in the hypoxic endothelium of coronary arteries of failed heart (Cheng et al., 2001; Moniotte et al., 2001; Michel et al., 2020), where they were demonstrated to be involved in adrenergic-induced vasodilation and re-vascularization, through the nitric oxide (NO) pathway (Dessy et al., 2004); 2) the increased NO production following β3-AR activation seems to exert a pro-angiogenic action as recently demonstrated in a model of limb ischemia induced by diabetes (Bubb et al., 2021); 3) in the 129S6 mouse strain, a breed with a predisposition to produce significantly higher levels of VEGF and to develop a particularly aggressive retinal neovascularization in response to hypoxia if compared with the C57BL/6J strain (Chan et al., 2005), β3-AR mRNA was massively upregulated, and the response to propranolol was insignificant (Chen et al., 2012). These data, including the discrepancy in different animal strains, explainable by their different genetic background (Filippi et al., 2012), suggest that β3-ARs could be actively involved in the angiogenic process.

The involvement of β3-ARs in ocular vascularization processes is still not well defined. On the one hand, in the C57BL/6J strain exposed to hypoxia, propranolol, but not a selective β3-AR antagonist, is effective in reducing retinal neovascularization (Martini et al., 2011), apparently suggesting a negligeable proangiogenic role of β3-ARs. On the other hand, recently, in human patients treated for 3 months with a selective β3-AR agonist a significant impact of choroidal vascularity has been shown, suggesting instead for this receptor a potential proangiogenic effect (Topcuoglu and Aslan, 2021). Certainly, many of these apparent contradictions might be explained by a more in-depth knowledge on the pharmacokinetic and pharmacodynamic properties of the molecules active on β3-ARs. In this regard, the majority of the molecules usually employed to agonize or antagonize β3-ARs show serious limitation of selectivity. In particular, the widely used antagonist SR59230A is not always selective for β3-ARs (Candelore et al., 1999), and can act as a partial agonist, or even as a full agonist (Hutchinson et al., 2005). On the other side, the widely used agonist BRL 37,344 shows an affinity for rodent β3-ARs 20-100 times higher than for human receptors (Vrydag and Michel, 2007), while a better specificity for human receptors has been reported by β3-AR agonists entered in clinical use such as mirabegron (Igawa and Michel, 2013). The functional role of β3-ARs in angiogenic processes has been further clarified by dedicated studies aimed at testing the actual involvement of β3-ARs in the hypoxia-driven retinal neovascularization. In mouse retinal explants β3-ARs were confirmed to be up-regulated by hypoxia, and to modulate the hypoxia-induced VEGF release through the activation of the NO signaling pathway (Dal Monte et al., 2013b). These results were corroborated by the observation that in the OIR model the activation of β3-ARs during the proliferative phase induces retinal vessel proliferation although at much lesser extent than in β1/2-AR knockout mice which are almost unresponsive to hypoxia mice (Dal Monte et al., 2015).

The lack of efficacy of β3-AR antagonism in the mouse strain responsive to propranolol does not exclude the possibility that the mouse strain unresponsive to propranolol in which β3-ARs are dramatically up-regulated, might help to uncover the possible role of β3-ARs in retinal vascularization. Whether in preclinical models β3-AR role would be uncovered, its therapeutical application against the progression of severe ROP unresponsive to propranolol would not imply a timely clinical translation. In fact, while molecules with acceptable selectivity against β3-ARs are currently available for animal studies (Manara et al., 1996; Candelore et al., 1999), drugs designed to selectively antagonize human β3-ARs have never been developed (Nagiri et al., 2021).

## New Perspectives to Prevent ROP Occurrence

So far, the greatest efforts in preventing the development of ROP have been oriented towards a defensive strategy, focused on the careful and appropriate use of oxygen. However, a detailed analysis of the most recent advances in understanding the pathogenesis of ROP, suggest new preventive perspectives.

The biological mechanisms by which HIF-1 regulates the vascularization during the intrauterine life are only partially known and understanding them more precisely could pave the way for even more daring consequences. The demonstration that β2-ARs blockade with propranolol uncouples the link between hypoxia and neovascularization (in IH/ROP patients or in the OIR model when administered during the proliferative phase), suggests the specular hypothesis that the stimulation of β2-ARs might uncouple the link between relative hyperoxia and vascular regression. Theoretically, all β-AR subtypes may participate to couple oxygen levels to retinal vascularization, but β2-AR agonism under hyperoxia might represent apparently the most intuitive strategy to prevent vascular regression. However, prolonged β2-AR activation usually leads to loss of agonism efficacy, a phenomenon defined as functional desensitization (Goral et al., 2011). In this respect, isoproterenol, an unselective β-AR agonist, when administered in OIR mice during the proliferative phase, reduced the retinal levels of VEGF and neovascular tuft formation similarly to propranolol (Dal Monte et al., 2012). If this is the case when agonizing β2-ARs during hyperoxia, then the possible complication of VEGF reduction would further accentuate hyperoxia-induced vascular regression, an effect opposite to what expected. Therefore, the perspective to face the vascular regression with β2-AR agonists is at first glance intriguing, but with uncertain results.

However, the demonstration in different hypoxic scenarios (OIR or ischemic hearth) that HIF-1α and β3-ARs are up-regulated and actively involved in promoting neo-vascularization suggests to focus the attention also on β3-ARs, which might be involved in this intermediary role. At the same time, hypoxia promotes a significant up-regulation of catecholamines, and therefore ligand and specific receptors are up-regulated under hypoxia (Dal Monte et al., 2012). Considering that HIF-1α, β3-ARs and catecholamines are coordinately activated in different hypoxic scenarios including the intrauterine environment, it is legitimate to imagine that an early exposure to high oxygen tension at birth might tentatively induce vascular regression through a coordinated action on HIF-1α, β3-ARs and catecholamines.

In this regard, it is important to note that in blood mononuclear cells, β3-ARs, which are up-regulated under hypoxia, become quickly down-regulated after oxygen re-exposure (Calvani et al., 2019), demonstrating that β3-ARs, as HIF-1α and VEGF, are inversely regulated by oxygen. Moreover, β3-ARs, which were recently identified in fetal ductus arteriosus, where they appear to participate to the patency maintenance, soon after delivery decreased in their levels, suggesting a possible direct regulation of β3-ARs expression by high oxygen levels (Pini et al., 2020). Whether in the retina, β3-AR expression would be demonstrated to be related to oxygen tension, premature exposure to high oxygen tension that induces retinal vessel regression would be tentatively related to β3-AR down-regulation in concomitance with lowered levels of HIF-1α and VEGF.

Whether the role of β3-ARs in retinal vascularization would be indisputably proven, then scenarios that currently appear futuristic might open up (Filippi et al., 2021). In particular, it would be possible to evaluate the hypothesis of counteracting the ischemic phase of the disease by intervening on β3-ARs through their selective activation with specific agonists (already available for children) (Keam, 2021). This would eventually prevent retina vessel regression irrespectively on hyperoxia with an efficacy similar to that already observed in preclinical models using PHD inhibitors (Sears et al., 2008; Hoppe et al., 2016), but without the complication of activating a plethora of target genes.

## Conclusion

In conclusion, the current therapeutic armamentarium for infants with ROP is unfortunately still limited to invasive interventions (laser phototherapy or anti-VEGF drugs) in a very advanced stage of ROP, while no effective therapy or preventive interventions are available in a precocious stage of the disease.

**FIGURE 2:**
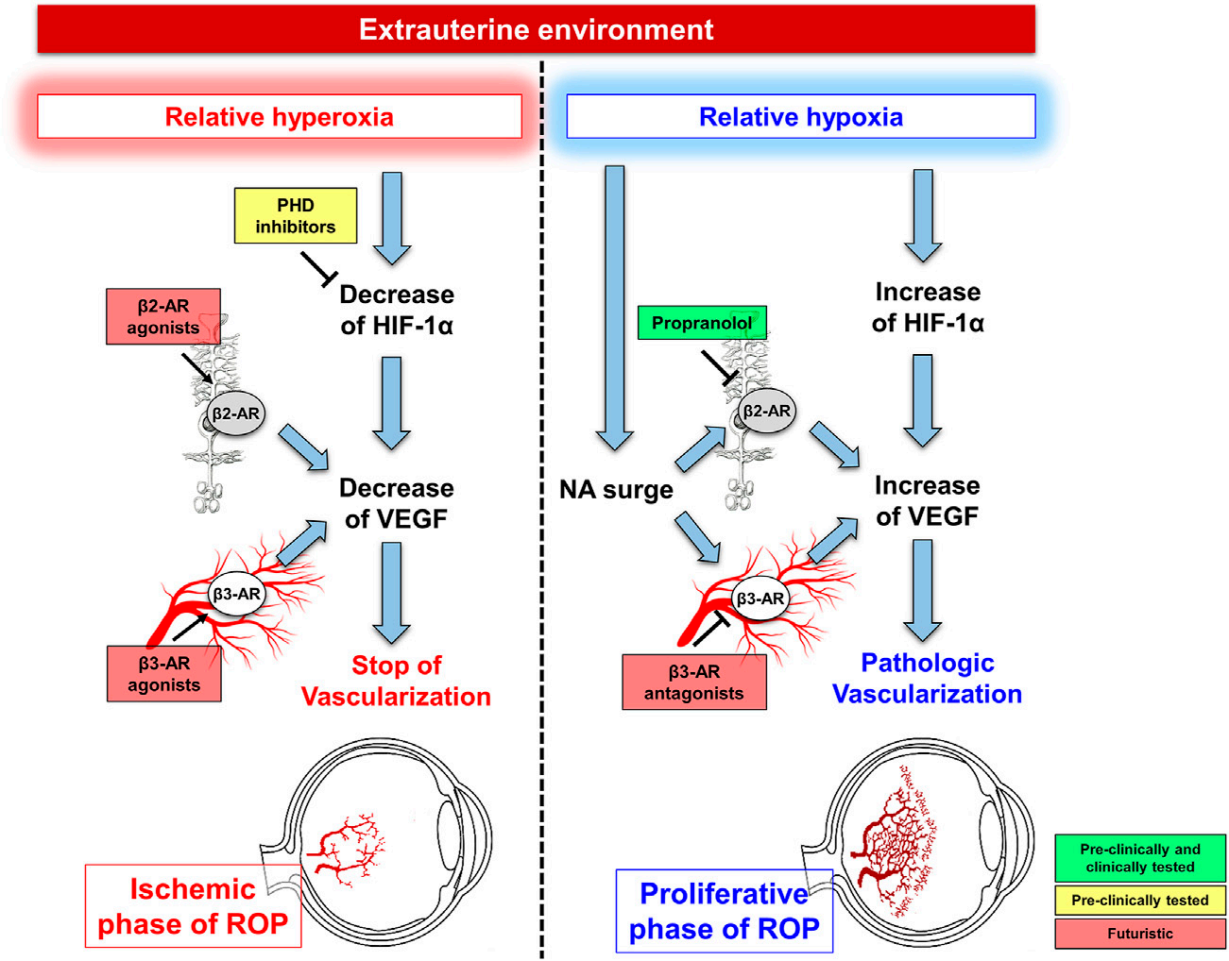
Current perspective and futuristic scenarios for ROP prevention and treatment. During the ischemic phase of ROP, futuristic are the hypotheses to prevent ROP occurrence through prolyl hydroxylase domain-containing proteins (PHD) inhibitors or β-adrenoceptor (β-AR) agonists aiming at preventing hyperoxia-induced vascular regression thus hindering the proliferative phase of ROP. The shift from hyperoxia to hypoxia is characterized by HIF-1α upregulation, which promotes VEGF production leading to retinal vessel proliferation. Concurrently, noradrenaline (NA) surge activates β2-ARs, expressed by Müller cells and β3-ARs, localized to endothelial cells, both participating to VEGF accumulation. Blockade of β2-ARs with propranolol is the goal of the current perspective to counteract ROP progression, while the futuristic approach of antagonizing β3-ARs deserves further investigations.

However, an intense research activity is currently underway in these years aimed at identifying new therapeutic and prophylactic strategies. Among these, the most fascinating strategy seems to be represented by the attempt to decouple the different oxygen levels from their effects on vascularization. The eventuality of treating infants with ROP through propranolol administration in order to decouple hypoxia from retinal neovascularization seems at hand. Instead, the perspective to pharmacologically decouple the exposure to a relative hyperoxia from vascular regression appears more futuristic (Figure 2).

## Abbreviations

HIF-1, hypoxia-inducible factor-1; IHs, infantile hemangiomas; NO, nitric oxide; NA, noradrenaline; SpO2, oxygen saturation; OIR, oxygen-induced retinopathy; PHDs, prolyl hydroxylase domain-containing proteins; ROP, Retinopathy of prematurity; VEGF, vascular endothelial growth factor; VLBW, very low birth weight; β-ARs, β-adrenoceptors.
